# Salt, blood pressure and cardiovascular risk: what is the most adequate preventive strategy? A Swiss perspective

**DOI:** 10.3389/fphys.2015.00227

**Published:** 2015-08-13

**Authors:** Michel Burnier, Gregoire Wuerzner, Murielle Bochud

**Affiliations:** ^1^Service of Nephrology and Hypertension, Department of Medicine, University HospitalLausanne, Switzerland; ^2^Institut Universitaire de Médecine Sociale et Préventive, University HospitalLausanne, Switzerland

**Keywords:** blood pressure, sodium, potassium, health strategies, hypertension, cardiovascular risk

## Abstract

Among the various strategies to reduce the incidence of non-communicable diseases reduction of sodium intake in the general population has been recognized as one of the most cost-effective means because of its potential impact on the development of hypertension and cardiovascular diseases. Yet, this strategic health recommendation of the WHO and many other international organizations is far from being universally accepted. Indeed, there are still several unresolved scientific and epidemiological questions that maintain an ongoing debate. Thus what is the adequate low level of sodium intake to recommend to the general population and whether national strategies should be oriented to the overall population or only to higher risk fractions of the population such as salt-sensitive patients are still discussed. In this paper, we shall review the recent results of the literature regarding salt, blood pressure and cardiovascular risk and we present the recommendations recently proposed by a group of experts of Switzerland. The propositions of the participating medical societies are to encourage national health authorities to continue their discussion with the food industry in order to reduce the sodium intake of food products with a target of mean salt intake of 5–6 grams per day in the population. Moreover, all initiatives to increase the information on the effect of salt on health and on the salt content of food are supported.

## Introduction

The role of sodium in human physiology has been recognized more than two thousand years ago, but the importance of maintaining an equilibrated sodium balance in order to maintain body volumes and blood pressure (BP) has been demonstrated essentially by the pivotal publications of Claude Bernard in the 1850's on the regulation of the ≪ *milieu intérieur* ≫ and the fantastic work of the physiologist Arthur Guyton and his studies on BP regulation and the kidney (Guyton et al., 1980). Today it is well-accepted that an excessive salt intake is deleterious for health mainly because a high salt intake favors the development of essential hypertension and its cardiovascular and renal complications (Whelton et al., 2012; He et al., 2013; Kotchen et al., 2013; Mozaffarian et al., 2014). For these reasons the WHO recommendations as well as those of many national medical societies encourage to lower salt intake in the population (World Health Organisation, 2003).

Despite the accumulation of experimental, epidemiologic and clinical data confirming a causal association between salt intake and the risk of developing hypertension and cardiovascular complications with recent data obtained in very large sets of population (Mente et al., 2014; Mozaffarian et al., 2014) the issue of salt intake in the population remains a matter of an intensive scientific and political debate which is now going on for several decades (He et al., 2013; Kotchen et al., 2013; Mente et al., 2014). Indeed, the answers to several questions remain highly controversial among the medical community. The first regards the ideal amount of salt that should be consumed by the population. The second concerns the pertinence of the proposed strategies to reduce salt intake: should one continue to promote a salt reduction in the entire population or would it be wiser to target individuals at high risk of health complications due to an excessive salt intake? At last could a diet too low in sodium represent a risk and eventually increase the mortality risk as suggested by some (Stolarz-Skrzypek et al., 2011; Graudal et al., 2014; Mente et al., 2014), but contested by others (Cook et al., 2014).

Recently a working group on Salt and Health has been created in Switzerland under the patronage of the Swiss Foundation of Cardiology and with the financial help of the Swiss Federal Health authorities. The goal of this group of local experts was to edit a position paper on salt and health in order to clarify the position of the various medical specialties to physicians and healthcare professionals and to support the national strategy on salt in Switzerland that could help preventing serious chronic diseases affecting adults as well as children. Thus, specialists in hypertension, nephrology, cardiology, pediatric, public health, geriatric, neurology, angiology, endocrinology, nutrition, obstetric, internal medicine and general medicine have been involved in the redaction of a position paper on salt. This document has been published on the site of the Swiss Foundation of Cardiology http://www.swissheart.ch/index.php?id=2458&L=1) in German, French and Italian. The present article is a reappraisal of this position paper in English in which the most recent literature and considerations have been added.

## Strategies to reduce salt intake and international initiatives

For the World Health Organization (WHO) and the United Nations reduction of salt intake in populations is considered as a health strategy with the best cost/effectiveness to prevent the development of non-communicable diseases such as cardiovascular and renal diseases (Beaglehole et al., 2011). For this reason, WHO has always supported national programs aiming at reducing the mean salt intake of the general population to a maximum of 5 g of sodium chloride (NaCl) or 2000 mg of sodium (Na). The World Hypertension League and the International Society of Hypertension also support the position of the WHO and together with the United Nations recommend to participating countries to lower salt intake of their population by 30% by 2025. In between several countries have established their own national strategies and published guidelines with more or less ambitious targets (He et al., 2010). Thus the United States and Great Britain recommend a salt intake of less than 6 g sodium chloride per day and British even have a target of 3 g NaCl until 2025 (He et al., 2010). In the countries of the European community, the goal is to lower salt intake by 16% within 4 years and Switzerland actively collaborates to the European Salt Action Network. This Network has actually recognized the necessity to evaluate the situation in each country, to define clear objectives, to develop an information concept for the public, to accelerate the collaboration with the industry and to establish a monitoring system.

**Figure 1:**
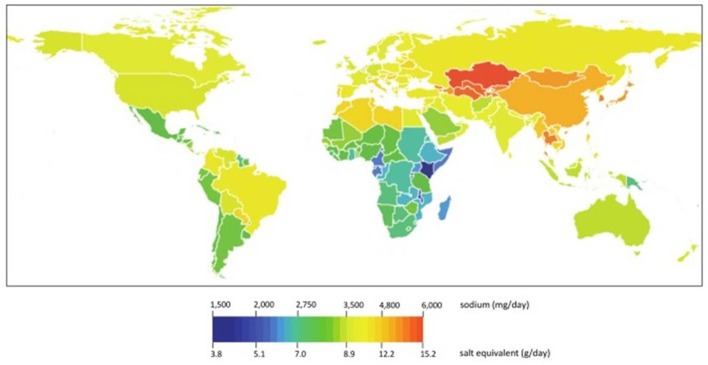
**Mean global sodium intakes in 2010, by country worldwide (from Powles et al., 2013)**.

A recent review of the multiple national initiatives has actually demonstrated that salt intake is relatively variable among countries but generally varies between 6 and 12 g/day in adults with a higher intake among men than women (Webster et al., 2011). These data were confirmed recently in large survey showing that salt intake exceeds the WHO recommendations in 181 of 187 countries around the world (Powles et al., 2013; Mozaffarian et al., 2014). (Figure 1) Depending on the local traditions and eating habits salt intake may be as high as more than 20 g NaCl per day. However, one should mention that the various techniques used to assess salt intake may also contribute to the observed variability as not all investigators used 24 h urine collections, the reference technique, to measure salt intake.

Today, six countries have reported positive results of their salt reducing strategy leading to a positive impact on the population health i.e Finland, Great Britain, France, Ireland, Japan, and recently Portugal (Polonia and Martins, 2009; Webster et al., 2011). In Finland, the first country to have organized an intervention to reduce salt intake at the national level, salt intake has decreased by 3 g/day between 1978 and 2002. During the same time period BP decreased significantly and cardiovascular mortality and stroke declined by 60% (Laatikainen et al., 2006). In Great Britain, between 2003 and 2011 salt intake decreased by 1.4 g per day and was associated with a significant decrease in BP and cardiovascular events but several other parameters improved simultaneously such as smoking, cholesterol and potassium intake (He et al., 2014). These examples demonstrate the feasibility of these strategies but also emphasize the difficulty to lower salt intake substantially over a short time period.

## The Swiss salt strategy

In Switzerland, the Federal Office of Public Health has elaborated a national strategy to reduce salt intake which is called Salt Strategy 2008-2012. This program has now been extended until 2016. The short-term goal of this program is to reduce salt intake of the Swiss population initially to less than 8 g/day and in the long-term to the recommended target of < 5 g/day. The first step of this national program was to assess salt intake in the population in the context of the Swiss Salt Study. The results of this study are available on the site: http://www.bag.admin.ch/suchen/index.html?keywords=Salt&lang=fr&site_mode=intern&nsb_mode=yes&search_mode=AND#volltextsuche (access verified in June 2015).

**Figure 2:**
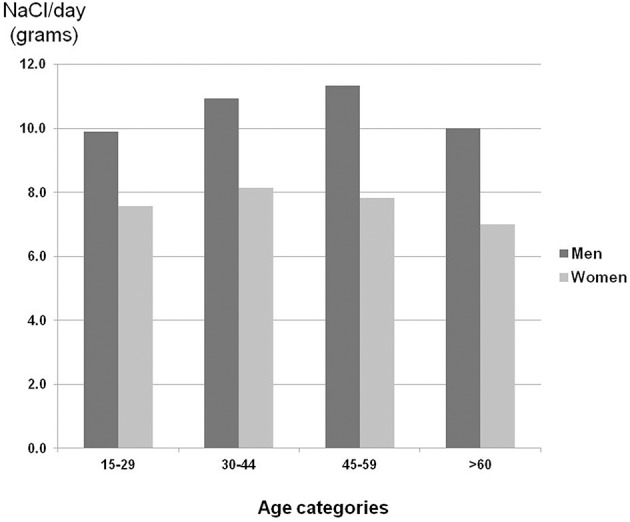
**Salt intake based on 24 h urinary sodium excretion in Switzerland according to age and sex**. Note that sodium intake is much lower in women and that there is a trend for a lower salt intake in the youngest and oldest age categories for both men and women.

In this national survey conducted in 2010 and 2011, 24 h urines were collected in 1447 subjects aged >15 years and randomly contacted throughout Switzerland taking into account the three linguistic regions (French, German, and Italian). Results show a mean salt intake of 7.8 g/day in women and 10.6 g/day in men with little variations between the regions. As shown in Figure 2, salt intake tends to be lower in the youngest and oldest age categories both in men and women. In this study only 21.6% of women and 6% of men had a salt intake in line with the WHO recommendations. These figures are quite similar to those reported in other surrounding European countries (Webster et al., 2011). BP was also measured in all participants and the prevalence of hypertension defined as a BP > 140/90 mmHg or taking antihypertensive drugs was 25.6% with a higher prevalence in men (32.3%) than in women (19.1%). Interestingly, although sodium intake was comparable in the 3 linguistic regions, significant differences in the prevalence of hypertension were observed between the 3 regions (higher prevalence in the German speaking area and lower prevalence in the Italian-speaking region). As reported in previous studies a weak correlation was found between urinary sodium excretion and BP but the correlation was stronger and significant in subjects aged >50 years with an increase in systolic BP of 0.51 mmHg for each gram of salt (*p* < 0.02).

The Swiss Salt Strategy shares many common points with other national programs in Europe. Thus, authorities have decided to establish a constructive dialog with the food industry and the food producers (bakers, butchers, cheese makers…) in order to reduce salt in dietary products based on a voluntary basis rather than on political constraints. There is also a strong will to improve the population information not only by explaining the potential risks of a salt excess but also by improving the labeling of products in order to ameliorate the ability to choose food products with a lower salt content. In parallel, several research projects were supported to test the feasibility of lowering the salt content of some basic products without affecting the taste and to investigate whether reducing salt in collective restaurants would be a pertinent approach.

## Health consequences of a high salt diet

The health consequences of an elevated salt intake have been described in numerous experimental and observational studies (for review, see Alderman et al., 1998; Beaglehole et al., 2011; Äijälä et al., 2015). Globally, studies have demonstrated that a high salt diet increases BP and thereby increases the risk of developing cardiac, cerebro-vascular, vascular and renal complications. Several large population studies including our Swiss Salt Study have demonstrated a positive association between BP and the habitual sodium intake (Frost et al., 1991; Elliott et al., 1996; Strazzullo et al., 2009; Joosten et al., 2014). The INTERSALT study was one of the first to demonstrate a lower prevalence of hypertension in populations with a low urinary sodium excretion (Elliott et al., 1996). In this study the age-associated increase in BP correlated closely with urinary sodium excretion suggesting that the aging phenomenon is linked to an increased salt sensitivity (Elliott et al., 1996). An association between BP and a high sodium intake has also been observed in children and adolescents (He and MacGregor, 2006). The link between salt intake and BP has been recently re-emphasized by the results of the Prospective Urban Rural Epidemiological (PURE) study in which more than 100,000 subjects were enrolled (Mente et al., 2014). In this study, two interesting observations were made. The first is that the association of estimated intake of sodium with BP was non-linear, a steeper association being found with a higher sodium intake. The second is that the association was most pronounced not only in persons consuming high-sodium diets but also in persons with hypertension, and older persons (Mente et al., 2014). Several other observational studies had actually reported a causal relationship between salt consumption and arterial hypertension and cardiovascular diseases in various populations with sometimes contradictory results (Alderman et al., 1998; He et al., 1999; Tuomilehto et al., 2001; Zhao et al., 2004; Geleijnse et al., 2007).

As far as complications are concerned, a high salt diet has been associated with an increased risk of stroke (Perry and Beevers, 1992), left ventricular hypertrophy (Kupari et al., 1994; Jin et al., 2009), proteinuria (Lambers Heerspink et al., 2012a), and progression of chronic kidney diseases (CKD) (Lambers Heerspink et al., 2012b) sometimes independently of the effect of salt on BP. Salt excess also limits the antihypertensive effect of drugs such as diuretics and blockers of the renin-angiotensin system (Lambers Heerspink et al., 2012b). Finally, a high sodium diet has been associated to an increased incidence of stomach cancer (Joossens et al., 1996), metabolic syndrome (Chen et al., 2009), obesity, an excessive consumption of soft drinks (He et al., 2008) and to an increased risk of developing renal stones (Cappuccio et al., 2000). Of note, many effervescent drugs have been reported to contain a high amount of sodium (George et al., 2013). A chronic consumption of these drugs may contribute to increase the cardiovascular risk of patients (George et al., 2013).

The impact of salt on BP and cardiovascular events has also been investigated in many interventional studies (1992; 1997; Cappuccio et al., 1997; Whelton et al., 1998; Sacks et al., 2001; Gates et al., 2004; He et al., 2009, 2013). The majority of these studies have shown that reducing salt intake has a positive effect on BP and on the incidence of cardiovascular and renal complications and this is reflected in the results of several meta-analyses and a Cochrane analysis (Midgley et al., 1996; Graudal et al., 2012; He et al., 2013). In general the relation is weak among individuals with a normal BP and probably a normal capacity to excrete sodium and significantly more marked in hypertensive and elderly patients characterized by a reduced ability to excrete sodium. According to Strazzullo's meta-analysis a reduction of salt intake of 5–6 g/d is associated with a decrease in systolic BP of 4–5 mmHg and diastolic BP of 1–3 mmHg (Strazzullo et al., 2009). Similar figures were obtained in the meta-analysis published by He et al. with a reduction of systolic BP of 5.4 mmHg and diastolic BP of 2.8 mmHg in hypertensive patients reducing their salt intake by 100 mmol/day, i.e., 5 g NaCl/day (He et al., 2013). Although apparently small these decreases in BP should be sufficient to induce a significant reduction of stroke and cardiovascular events as a modest reduction of 3 g/day has been estimated to substantially reduce cardiovascular events (Bibbins-Domingo et al., 2010).

## Salt consumption in children

Salt intake should not be severely restricted during pregnancy because sodium is necessary for the volume expansion of pregnant women. During the first years of the life and particularly in very small children, a positive sodium balance is necessary to promote an adequate body growth. Moreover, newborns and premature babies are particularly exposed to renal sodium losses. Therefore, they need a sufficient intake. Of note, breastfeeding and specific newborn food preparations contain sufficient amounts of salt to cover the needs. In adolescents, a high salt intake has the same consequences as in adults i.e., an increase in BP and weight gain (He and MacGregor, 2006) which may have an impact on BP level in adulthood. In terms of healthcare policy these issues are very relevant as an elevated BP in children and adolescents is often associated with a high BP during adulthood. In addition, the taste and eating habits are defined during childhood. Thus children that have been used to eat high salt meals will continue to prefer salty meals as adults. At last, in children and adolescents salt is often associated with a high consumption of soft drinks and sugar-containing drinks which are major causes of overweight and obesity (He et al., 2008).

## A strategy for high risk groups or for the entire population? the concept of salt sensitivity

The BP response to changes in sodium intake varies enormously among individuals. Indeed, whereas some subjects can eat very high amounts of salt without any modification of their BP, others experienced a significant rise in BP with a sodium-rich diet. This indicates that the BP response to changes in dietary sodium is heterogeneous and this observation has led to the development of the salt-sensitivity concept (Kawasaki et al., 1978; Luft et al., 1991). The validity of this concept has been challenged many times but the clinical observation remains valid. One concern has been the arbitrary definition of salt sensitivity which had been defined by Weinberger et al. as an increase in BP of more than 10% on high sodium diet (Weinberger, 1993). Thereafter many different definitions and approaches to characterize salt sensitivity have been proposed in order to be more accurate. (Lungershausen and Howe, 1994). The arbitrary dichotomization of the population has also been questioned. Indeed, the distribution of salt-induced changes in BP is normal and there is no evidence for a bimodal distribution. Therefore, a salt-sensitive population could not be clearly defined. At last there have been always some concerns about the reproducibility of the determination of salt sensitivity (Zoccali et al., 1996; Chiolero et al., 2001). Despite these criticisms, salt sensitivity of BP remains a topic of clinical interest because of its public health impact as a high salt sensitivity has been associated with an increased risk of cardiovascular events and death (Morimoto et al., 1997).

Salt-sensitivity is relatively common in the population. Thus earlier studies have demonstrated that 30–50% of hypertensive patients and 10–20% of normotensive subjects are salt-sensitive (Weinberger et al., 1986). Certain groups of patients are more sensitive to salt. This is the case for example of subjects or patients of African origin and elderly patients. The prevalence of salt-sensitivity may have increased in the last decades with the rising prevalence of obesity, metabolic syndrome, type 2 diabetes, and CKD. Indeed, studies have demonstrated that obesity and metabolic syndrome are associated with an increased salt-sensitivity (Chen et al., 2009). This also clearly the case for patients with CKD and a limited renal function (Kimura et al., 2010). Interestingly if one considers the actual prevalence of obesity, metabolic syndrome, diabetes, CKD, hypertension, advanced age and subjects of African origin, the salt-sensitive population may well represents more than 50% of the entire population and this may actually justify a global salt strategy rather than a strategy focused on high risk patients groups.

The salt sensitivity of BP is mediated by several mechanisms involving the vessels, the brain, the kidneys and of course genetics factors (Cowley, 1997; Weinberger, 2006a; Sanada et al., 2011). Seminal experimental models such as those developed in the rat by Dahl and collaborators (Dahl et al., 1962; Mattson et al., 2008) and the recent description of several monogenic forms of essential hypertension linking the development of hypertension to a renal tubular defect limiting the excretion of sodium have clearly demonstrated the crucial roles of genetic factors and renal sodium handling in the salt-sensitive phenotype (Lifton, 1996; Ji et al., 2008). Yet, many other factors have an impact on the salt sensitivity of BP. Among them one has to mention the hormonal profile (renin-angiotensin-aldosterone system, sympathetic nervous system, sexual hormones…) and importantly renal function and the renal capacity to excrete sodium (Chiolero et al., 2001; Weinberger, 2006b; Pechere-Bertschi and Burnier, 2007).

The heterogeneity of the BP response to changes in salt intake and the concept of salt-sensitivity has been the basis of the question regarding the most adequate strategy to pursue in order to reduce the negative clinical impact of a high sodium consumption. Should one restrain sodium intake in the entire population or only in salt-sensitive subjects and patients who are at higher risk of developing hypertension and cardiovascular and renal complications? For reasons of cost/effectiveness all national and international prevention programs have decided to build a strategy based on a global reduction of sodium consumption in the entire population. This decision is based on several observations. First, it is difficult to differentiate salt-sensitive from salt-resistant individuals in clinical practice as there is no easy tool to diagnose salt-sensitivity. Second, a modest reduction in salt intake should be beneficial for all individuals including young normotensive salt-resistant subjects whose phenotype might actually change as they become older and loose part of their capacity to excrete sodium with the renal aging process. At last it seems important to sensitize the entire population to the effects of salt and get them used to eat a salt-limited diet if possible already during childhood and adolescence. Some countries have actually combined the two strategies. Thus national organizations have included both approaches in their recommendations. For example, the American Institute of Medicine proposes a target salt intake of < 6 g NaCl /day in the general population but a lower target (< 3.8 g/day) in some groups of subjects such as individuals older than 50 years, hypertensive, diabetic or patients with renal diseases and subjects of African-American origin (Institute of Medicine; Strategies to reduce sodium intake in the United States, April 2010) (www.iom.edu/Reports/2010/Strategies-to-Reduce-Sodium-Intake-in-the-United-States.aspx). In Switzerland and most European countries, no specific recommendation is proposed for high risk groups despite their greater salt sensitivity.

## Is there a risk associated with a low salt diet

**Figure 3:**
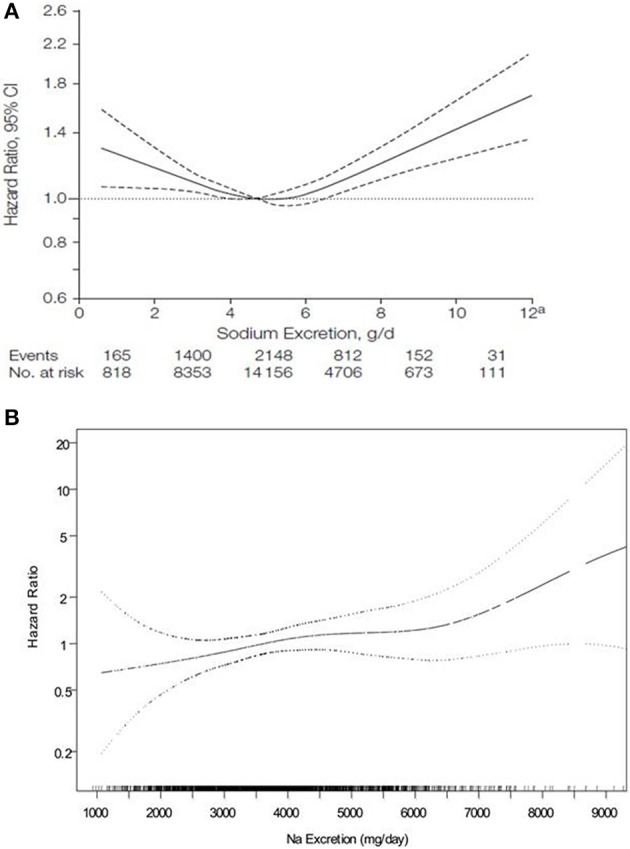
**Relationship between urinary salt excretion and the hazard ratio for cardiovascular events or death according to O'Donnell (A) suggesting a U-shape curve and according to Cook RN suggesting a linear relationship (B) (from O'Donnell et al., 2011; Cook et al., 2014)**.

In 2011, two observational studies were published simultaneously which provided intriguing and challenging new findings in clear opposition with most previously published studies. The first study was conducted by Stolarz-Skrzypek and Staessen's group (Stolarz-Skrzypek et al., 2011). They assessed whether 24-h urinary sodium excretion predicts BP and health outcomes in a prospective population study, involving 3681 participants without cardiovascular disease at baseline who were members of families that were randomly enrolled in the Flemish Study on Genes, Environment, and Health Outcomes (1985–2004) or in the European Project on Genes in Hypertension (1999–2001). Of 3681 participants without CVD, 2096 were normotensive at baseline and 1499 had BP and sodium excretion measured at baseline and last follow-up (2005–2008). Surprisingly, during the 7.9 years median follow-up, neither the risk of hypertension nor the risk of cardiovascular disease was related to urinary sodium excretion. In contrast, a low sodium excretion was associated with a higher cardiovascular disease mortality (Stolarz-Skrzypek et al., 2011). A rather similar observation was made by O'Donnell et al. who looked at the association between estimated urinary sodium and potassium excretion and cardiovascular events in patients with established cardiovascular disease or diabetes mellitus who participated in the Ongoing Telmisartan Alone and in Combination with Ramipril Trial (ONTARGET) and the Telmisartan Randomized Assessment Study in ACE-I Intolerant Patients with Cardiovascular Disease (TRANSCEND) trials (O'Donnell et al., 2011). In this very large study population of 28,880 participants, compared with a baseline sodium excretion of 4–5.99 g/day, sodium excretion of greater than 7 g/day was associated with an increased risk of all cardiovascular events, and a sodium excretion of less than 3 g daily was also associated with increased risk of cardiovascular mortality suggesting a U-shape curve of the relationship with an increased risk both at a low and at a high sodium intake (Figure 3A) (O'Donnell et al., 2011). Very recently, a similar observation was reported in a meta-analysis including most recent studies (Graudal et al., 2014) and in the PURE study and its 105,000 participants (Mente et al., 2014).

These studies have been criticized for their methodology regarding particularly the assessment of urinary sodium excretion (Whelton et al., 2012). This was the case for the PURE study and the first O'Donnell paper which did not use 24 h urine collections. There are also some doubts on whether a single measurement can reliably predict mortality over a prolonged follow-up period because of the variability of sodium intake. Nonetheless, these observations tend to confirm that an excessive salt intake (>15 g/day) is detrimental for health but do not support the recommendations to lower salt intake below 10 g/day. Interestingly, even in very high risk patients such as patients with CKD, it remains difficult to demonstrate that salt reduction reduces the risk of cardiovascular events and the decline in renal function although clear benefits on BP and proteinuria are consistently observed (McMahon et al., 2015).

In order to have a balanced view, one has to mention that several other studies with perhaps a better methodology have confirmed the clinical benefits of lowering salt intake (Äijälä et al., 2015; Merino et al., 2015). In contrast to the U-shape curve between urinary sodium excretion and cardiovascular events published by O'Donnell, Cook et al. found a linear relationship between the hazard ratio for cardiovascular events and urinary sodium excretion at all levels of sodium excretion in 2275 participants not in a sodium reduction intervention having participated in the THOP1 and THOP2 trials with 10–15 years follow-up (Cook et al., 2014) (Figure 3B).

Thus, from the data available today, the American Institute of Medicine has taken the following conclusions:

1. There is good evidence that a high salt consumption is positively correlated with a higher risk of cardiovascular events.
2. The evidence regarding the overall effects of salt on health are not yet coherent with the efforts made to lower salt intake to less than 3.75 g/d.
3. There is no strong evidence to restrict sodium intake more drastically in some sub-groups of patients differently from the entire population.

If a low salt diet is indeed associated with an increased incidence of cardiovascular events and mortality, the important question is what is the mechanism? Is this due to the low sodium intake *per se* or to one or more concomitant factors that might affect patients' survival? One might also question whether the low urinary sodium excretion observed in these patients is a consequence rather than a cause of their higher risk of cardiovascular complications. Indeed, in population studies, the number of subjects consuming less than 2 g of sodium is quite low. Thus intakes of this low level would seem likely to be a marker of ill health and frailty leading to a higher mortality. Today, the hypothesis of a deleterious effect of a low sodium diet is not scientifically proven. Adequately designed intervention studies should be done to answer this question. Moreover, a careful examination of the clinical context of subjects excreting a small amount of sodium every day should be done. Indeed it is well possible that a low sodium excretion is just a marker of risk and confounding factors may have played an important role. For example excessive doses of diuretics in heart failure, cirrhotic or diabetic, or CKD patients may increase their risk of complications (Paterna et al., 2008; Ekinci et al., 2011; Thomas et al., 2011). Yet, other hypotheses have been proposed to explain the U-shape relationship. The first is that a low sodium consumption is associated with a marked compensatory stimulation of the renin-angiotensin-aldosterone and of the sympathetic nervous system in order to maintain BP (Graudal et al., 2012). Several experimental and clinical studies have indeed shown that an activation of these hormonal systems is associated to the development of target organ damages such as left ventricular hypertrophy, vascular damages, proteinuria and a decrease in renal function. In these situations, sodium has a catalytic effect to augment the deleterious effects of angiotensin II and aldosterone and catecholamines (Brunner et al., 1972; Gavras et al., 1976; Brilla and Weber, 1992; Pimenta et al., 2008; du Cailar et al., 2010). However, one has to remember that these hormones have much less deleterious effects when subjects or animals are on a low sodium diet.

Another possible mechanism is the impact of a low sodium diet on serum cholesterol. Indeed, higher levels of serum cholesterol have been reported in subjects and patients with a low urinary sodium excretion (Harsha et al., 2004; Graudal et al., 2012) or on a low sodium diet as in the DASH trial (Sacks et al., 2001). An effect on lipid metabolism could actually increase cardiovascular events and mortality.

An excessive restriction of sodium in the diet may have several other clinical inconvenients. In Alpine countries such as Switzerland, iodine insufficiency is common and iodine supplementation is done by adding iodine to the kitchen salt. Recently, an adaptation of the iodine content of salt has occurred in Switzerland based on the results of the Swiss Salt Study (Haldimann et al., 2015). Reducing salt intake in the population may further aggravate the iodine status of patients. In patients on a low salt diet, there is also an increased risk of volume depletion and hypotension in case of acute dehydration or diarrhea. In very elderly, reducing sodium in the food may alter the food taste and increase the risk of malnutrition (Zeanandin et al., 2012). Thus, in elderly patients eating small amounts of food, sodium intake should probably not be reduced.

## Recommendations of the Swiss specialists

Today, the recommendations of the Swiss Society of Hypertension and the Swiss Society of Cardiology are in agreement with those of European societies which recommend a limitation of salt intake to less than 6 g/day for everybody. Several Swiss medical societies (nephrology, neurology, diabetology, pediatry, public health) do not have any specific recommendations on salt but they tend to follow the WHO guidelines or the recommendations of the cardiologists. The Swiss strategy also emphasizes the need to focus particularly on patients who have a high risk of developing arterial hypertension or cardiovascular complications i.e., patients with mild hypertension with or without target organ damage, subjects with a family history of hypertension, elderly patients and patients with chronic kidney and/or cardiac diseases, metabolic syndrome, diabetes or obesity. This approach should have a positive impact on the prevalence of hypertension in Switzerland which reaches 26% of the general population in Switzerland and more than 50% of Swiss older than 60 years. A modest reduction of salt intake should also contribute to decrease cardiovascular complications such as stroke, congestive heart failure and renal insufficiency.

The Societies also acknowledge that salt reduction is one of the approaches known to be effective in reducing cardiovascular events and mortality in Switzerland and that other strategies such as increasing potassium intake with fruits and vegetables, regulating calories intake and limiting the consumption of sweets and fat to prevent overweight and obesity should also be implemented.

In this context, the position and the proposal of Swiss scientific societies are the following:

1. At the national level, they invite the authorities to intensify their efforts to implement the Salt Strategy 2013–2016 and to mobilize necessary resources to this purpose. This strategy aims at reducing salt consumption at the population level below 8 g/day within 4 years and below 5 g/day in the long-term.
2. Societies will collaborate with the authorities to obtain sufficient financial support to favor research and to establish an efficient monitoring of salt consumption in Switzerland.
3. Societies will increase their engagements in the information of physicians and patients on the risks of a high salt diet, on the means to promote a reduction of salt intake and on the potential clinical benefits of lowering salt intake.
4. They will support all the efforts made to improve the information of consumers with the development of better labeling of food products as far as the salt content is concerned.
5. Scientific societies will also support the authorities in their efforts to convince the food industry to progressively lower that salt content of their products in order to achieve the goals of the Salt Strategy.

## Members of the working group on salt and health

M. Burnier (president), S. Aepli, M. Arnold, M. Bochud, D. Conen, P. Erne, D. Hayoz, C. Henzen, T. Junker, F. Muggli, T. Münzer, A. Pechère-Bertschi, F. Riesen-Christen, S. Schlüchter, V. Sibalic, G. Simonetti, D. Surbeck, P. Suter, B. Weil.

The original position paper was supported financially by the Swiss Federal Office of Public Health.

### Conflict of interest statement

The authors declare that the research was conducted in the absence of any commercial or financial relationships that could be construed as a potential conflict of interest.
